# Optical Properties of Electrospun Nanofiber Mats

**DOI:** 10.3390/membranes13040441

**Published:** 2023-04-18

**Authors:** Tomasz Blachowicz, Andrea Ehrmann

**Affiliations:** 1 Center for Science and Education, Institute of Physics, Silesian University of Technology, 44-100 Gliwice, Poland; 2 Faculty of Engineering and Mathematics, Bielefeld University of Applied Sciences, 33619 Bielefeld, Germany

**Keywords:** luminescence, sensing devices, absorbance, spectrometer, spectrophotometer, photocatalyst, UV/Vis

## Abstract

Electrospun nanofiber mats are usually applied in fields where their high specific surface area and small pore sizes are important, such as biotechnology or filtration. Optically, they are mostly white due to scattering from the irregularly distributed, thin nanofibers. Nevertheless, their optical properties can be modified and become highly important for different applications, e.g., in sensing devices or solar cells, and sometimes for investigating their electronic or mechanical properties. This review gives an overview of typical optical properties of electrospun nanofiber mats, such as absorption and transmission, fluorescence and phosphorescence, scattering, polarized emission, dyeing and bathochromic shift as well as the correlation with dielectric constants and the extinction coefficient, showing which effects may occur and can be measured by which instruments or used for different applications.

## 1. Introduction

Electrospinning can be used to produce very fine fibers, typically in the range of some ten to some hundred micrometers, from a polymer solution or melt [1,2,3]. Usually, a syringe with a fine needle or a polymer-coated wire or rotating drum enable introducing the polymer solution into a strong electric field, which forces the polymer droplets to fly from the high-voltage electrode to the grounded collector [4,5,6], while several other techniques are also available and enable faster spinning, or producing special fibers, such as core-shell fibers [7,8,9]. The nanofibers deposited on the collector are usually arbitrarily oriented, but different techniques such as a fast-rotating collector or introduction of conductive or dielectric areas in the collector can be used to gain a certain amount of orientation of the fibers [10,11,12]. The diameters of these nanofibers can have a broad distribution, while several studies concentrated on optimizing the electrospinning process so that this distribution is narrowed [13,14,15]. Furthermore, it is possible to give the electrospun nanofiber mats diverse physical or chemical properties, i.e., electrical or magnetic properties [16,17,18], by blending polymers or adding nanoparticles to the spinning solution [19,20,21].

All these aspects will influence the optical properties of nanofiber mats. Naturally, the structural properties of nanofiber mats will influence their optical properties, not only by the fiber diameter distribution, but also by defects [22]. As usual in polymers, the strain in the fiber surfaces will influence the absorption spectra of the nanofibers [23]. Tebyetekerwa and Ramakrishna describe how strain, deformations, electrical-charge storage, or even doping of electrospun nanofibers could be investigated optically, applying different methods such as photoluminescence (PL), absorbance, or polarization measurements [24]. On the other hand, it is possible to tailor the optical properties of nanofiber mats, e.g., to produce perovskite-embedded CsPbX_3_ nanofiber mats with different polymers which show a high photoluminescence intensity and can thus be used in highly luminescent white LEDs [25].

Measurements of the optical properties of such nanofiber mats are usually performed by spectrophotometric methods, often applying a UV-visible (UV/Vis) spectrometer, besides other techniques such as diffuse reflectance spectroscopic (DRS) measurements, etc. [26,27,28]. However, many more specialized methods are available and will be described in the next sections.

On the other hand, the impact of the environment on the optical properties of nanofibrous membranes makes them suitable as sensing devices, e.g., for toxicants, pH value, temperature, acids, volatile organic compounds, etc. [29,30,31,32,33].

This review describes the different optical properties of electrospun nanofiber mats, measurement techniques, and potential applications, concentrating on the visible light spectrum and partly directly neighboring parts of the spectrum, where the latter is measured with the same instrument, e.g., a UV/Vis spectrometer.

## 2. Absorption/Absorbance

The absorption and the absorbance characterize the ability of a material to absorb light. The absorbance *Abs* is defined as $Abs={log}_{10}(P_{in}/P_{trans})$ with the incident radiant power $P_{in}$ and the transmitted radiant power $P_{trans}$, or, as it is typically measured, $Abs={log}_{10}(P_{trans}^{ref}/P_{trans}^{sample})$ with the transmitted radiant powers $P_{trans}^{ref}$ of a blank reference and $P_{trans}^{sample}$ of the sample under investigation [34]. Absorption coefficients are sometimes given as numbers, sometimes with the unit m^−1^, so that care must be taken comparing different studies [34].

One of the fields in which broadband light absorption is important is light harvesting, e.g., for solar steam generation [35,36,37]. Liu et al. produced an Ag@MXene/poly(acrylonitrile) (PAN) nanofiber mat, which they used as a solar evaporator, and found that the combination of the plasmonic Ag nanoparticles (NPs) and the MXene nanosheets resulted in a large sunlight absorption of 93% along the whole solar spectrum as well as catalytic degradation of nitro compounds and antibacterial properties [38]. Using a folded origami structure, they produced a solar evaporator, which was largely independent from the angle of the impinging sunlight and could evaporate water with up to 2 kg/(m^2^ h).

Optical sensors can also be based on changes in absorption [39,40,41]. Abedalwafa et al. prepared colorimetric biosensors from electrospun nanofiber membranes with aptamer-conjugated Ag NPs [42] (aptamers are short, single-stranded DNA or RNA which show high selectivity for binding to well-defined targets), which they used to optically detect kanamycin as a model analyte by detecting a color change from pink to white.

**Figure 1:**
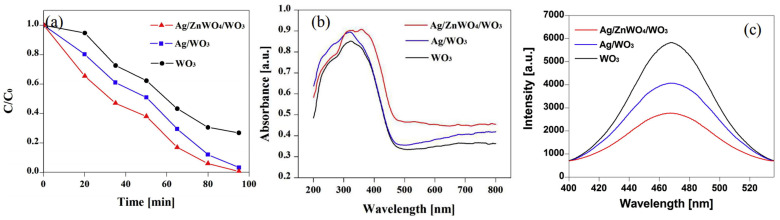
(**a**) Photocatalytic decomposition of methylene blue with WO_3_, Ag/WO_3_, and Ag/ZnWO_4_/WO_3_ composites nanofibers, (**b**) UV/Vis diffuse reflectance spectra, and (**c**) photoluminescence spectra of as-prepared samples. From [50], copyright (2019), with permission from Elsevier.

A broad research area is related to photocatalytic degradation, which can be improved if the absorption of the catalyst can be found in the visible light range, i.e., in the largest part of the sunlight spectrum [43,44,45]. For this, Jian et al. prepared La-doped ZnO nanofibers by electrospinning and subsequent calcination [46]. They showed that a small amount of La could reduce the relatively large band gap of ZnO, enabling more absorption of visible light. This, in turn, resulted in better photocatalytic degradation of the model dye Rhodamine B. Similarly, Baylan and Yildirim used manganese to dope ZnO nanofibers and reported a red-shift of the band edge so that larger wavelengths, i.e., smaller energies of the arriving light are necessary for photocatalytic activities, correlated with improved photocatalytic degradation activity as tested for methylene blue model dye [47]. Lim et al. used Bi_2_O_3_ nanofibers for photocatalytic tests and showed that by combining the two phases, α-Bi_2_O_3_ and β-Bi_2_O_3_, the band gap could be tailored, resulting in the possibility to optimize the degradation of Rhodamine B [48]. Another way to improve the photocatalytic degradation of methylene blue was chosen by Aghasiloo et al. who prepared highly porous TiO_2_ nanofibers by electrospinning in a humid environment [49]. The increased light absorption due to the high specific surface area also significantly increased the photocatalytic degradation efficiency. A more complex composition of nanofibers was chosen by Wei et al. who prepared Ag/ZnWO_4_/WO_3_ composite nanofibers, which showed a clearly higher absorption edge compared to Ag/WO_3_ or WO_3_ solely, as depicted in Figure 1b, resulting in higher photocatalytic degradation of methylene blue (Figure 1a) [50]. Here, in addition, the reduced photoluminescence (PL) intensity of the Ag/ZnWO_4_/WO_3_ nanofibers (Figure 1c) was mentioned as another factor influencing the photocatalytic activity, showing that Ag/ZnWO_4_/WO_3_ nanofibers accelerated the separation of the photogenerated electron-hole pairs. Combining Ga_2_O_3_/ZnO/WO_3_ heterojunction composite nanofibers, Zhang et al. could also improve the photocatalytic degradation of Rhodamine B as compared to WO_3_ or ZnO/WO_3_ nanofibers, which was attributed to enhanced optical absorption and suppression of carrier recombination, and the S-scheme heterojunction interface built by the contact of Ga_2_O_3_, ZnO, and WO_3_ [51]. Lu et al. found improved photoreactivity of TiO_2_ nanofibers assembled from nanosheets, which further increased the specific surface area and correspondingly the photocatalytic oxidation of acetone [52].

In addition to this often-mentioned area of application, the absorbance of nanofiber mats is also measured for basic research, in this case not necessarily showing a red-shift of the UV/Vis absorbance. Sharma et al., e.g., produced polyvinyl pyrrolidone (PVP)/poly(ethylene oxide) (PEO) nanofibers filled with PbS nanoparticles and found a significantly increased absorption with increasing PbS content, combined with a blueshift of the reflectance onset, besides tenability of the band gap by the size of the PbS nanoparticles [53].

While absorbance measurements are, as mentioned in this section, often used to tailor the absorbance edge towards higher wavelengths to reach better photocatalytic properties or for similar applications, these measurements can also be used to investigate the optical band gap of materials, as described in the next section.

## 3. Optical Band Gap Investigation

**Figure 2:**
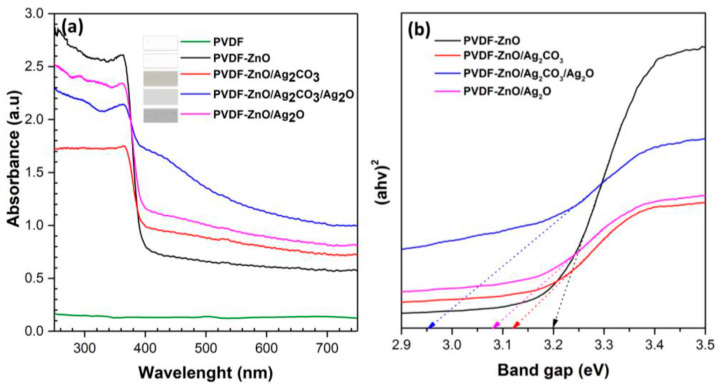
(**a**) Absorbance spectra, (**b**) band gap determination by drawing the line at (αhυ)^2^ of the electrospun nanofibers. For polyvinylidene fluoride (PVDF), no band gap could be calculated since the absorbance was too low. From [55], copyright (2019), originally published under a CC-BY license.

The optical band gap of semiconductors is usually detected from absorption spectra by using Tauc plots. The band gap energy can be estimated according to the Tauc formula $\left(\alpha h\nu \right)=A{\left(h\nu -E_{g}\right)}^{n/2}$ with the absorption coefficient α (proportional to the Kubelka–Munk function $F\left(R\right)={\left(1-R\right)}^{2}/2R$ with the reflectance R of the sample [54]), the photon energy hν, a constant A, the band gap $E_{g}$, and an integer n, which is 1 for direct and 4 for indirect band transitions [55]. As shown in Figure 2, the band gap can be calculated from plotting ${\left(\alpha h\nu \right)}^{2/n}$ versus the energy of the absorbed light, here for direct transition semiconductors, and the band gaps are directly found by extrapolating the linear parts of the spectra towards the *x*-axis [55]. In the case of indirect band gaps, the *y*-axis is ${\left(\alpha h\nu \right)}^{1/2}$ instead of ${\left(\alpha h\nu \right)}^{2}$ in the case of direct band gaps [56,57]. The graphs often show a doubled minimum or similar unexpected features if the wrong *y*-axis is chosen, while it is also possible to use both plots for immiscible blends with direct and indirect band gaps [56,57].

Using this technique, Matysiak et al. characterized the different blends of poly(acrylonitrile) (PAN) with the conductive polymers polypyrrole (PPy), polythiophene (PT), and polyaniline (PAni) and found a reduction in the band gap of pure PAN (4.08 eV) to minimum 3.77 eV for composite nanofibers containing 3% PAni [58]. Interestingly, Bayan et al. even found a significant difference in the band gaps of hollow core PAni and bulk PAni, which they used to improve the charge transport in the buffer layer of organic solar cells [59]. Oppositely, band gap energies of different disordered and ordered TiO_2_ nanofibers and nanotubes were identical within measurement accuracy, as shown by Wang et al. [60].

Sabzehmeidani et al. showed a strong band gap shift of CeO_2_/CuS composite nanofibers as compared to CeO_2_ nanofibers, which was favorable for visible light-induced photodegradation of methylene blue [61]. Similarly, Safartoobi et al. found different band gaps for Cu_(1−x)_Mn_x_Fe_2_O_4_ nanofibers with different x between 0 and 0.75 [62]. Gea et al. blended ZnO nanofibers with Ag and/or graphene oxide (GO) and found a reduction in the band gap from 2.98 eV for pure ZnO to 2.75 eV for ZnO-Ag-GO nanocomposites [63]. Similarly, SnO_2_ nanowires in pure form or as composites with PVP showed band gaps varying with structure and material blend [64,65].

## 4. Transmission

While the optical transmission through a sample is often calculated from absorbance measurements (and vice versa) by Lambert–Beer law Abs=−log⁡(T), with the transmission T defined as the fraction of light intensity visible behind the sample [66]. While it is usual to give the transmission in percent, it is mathematically impossible to have a unit in the argument of a logarithm, although this is also often found in the literature. As an example, an absorbance of 1 is correlated with a light transmission of 10%, while an absorbance of 2 means a light transmission of 1%.

Although both these values seem to be clearly correlated in theory, this law may cause practical problems, e.g., if reflection and scattering in the sample have to be taken into account, which are part of the extinction (cf. Section 5), but not of the absorbance, or if too high values of the absorbance (typically higher than 1.5) are measured [66]. A very detailed description of these and other typical practical measurement errors is given in [66].

In addition to these potential problems of calculating transmission from absorbance measurements, the literature research showed clearly that transmission measurements are mostly performed for different purposes, which is why this optical property is described in a separate section. Mostly, high transmission values are aimed at [67,68], e.g., in case of nanofibrous photodetectors based on transparent *p-n* junctions [69], transparent nanofiber-reinforced hydrogels for sensing or light-conducting applications [70,71], or solar-reflecting, infrared-transmitting nanofiber mats which block solar heating, but enable radiative cooling in the infrared [72].

**Figure 3:**
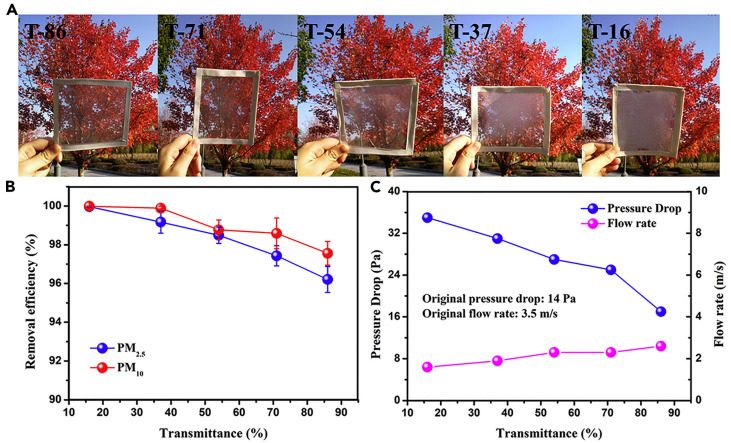
(**A**) Photographs of PDMS/PMMA-chitosan transparent air filters at different transparencies; (**B**) PM_2.5_ and PM_10_ removal efficiency; and (**C**) pressure drop and flow rate of transparent filters at different transmittances. From [74], copyright (2019), originally published under a CC-BY license.

Liu et al. investigated nanofibrous polyurethane (PU) electret window screens used for air filtration and found transmission values of approx. 5–50%, depending on the wavelength and on the areal density of the nanofiber mats [73]. Similarly, Liu et al. prepared transparent electrospun particulate matter filters from superhydrophobic PDMS/PMMA fibers and found an optical transmittance of 16–86% combined with high removal efficiency, as depicted in Figure 3 [74]. For the same purpose, Wang et al. showed electrospun filters with high transparency and improved filtration efficiency due to a different nanofiber distribution in the membrane, reached by a modified electrospinning process [75]. Similarly, Liang et al. demonstrated a highly transparent polyurethane nanofibrous air filter for fine particulate matter [76].

Another application of transparent nanofiber mats is given by edible films used for food packaging, which is why Ebrahimi et al. developed transparent gluten films containing nanofibers in which the nanofibers reduced the transparency, but at the same time eliminated the undesired yellowish color of the pure gluten films [77]. On the other hand, Feng prepared nanofiber mats from poly (lactic acid) (PLA) with different amounts of TiO_2_, which were highly intransparent at different UV wavelengths as well as at 600 nm, while the pure PLA film showed a transmission around 67% [78].

Finally, it should be mentioned that the optical property of transparency is correlated with the electrical sheet resistance and other electrical properties [79,80], which will be discussed subsequently.

## 5. Dielectric Constant and Index of Refraction

While the complex dielectric function is defined as $\varepsilon_{r}\left(\omega \right)=\varepsilon^{\prime }\left(\omega \right)+i\varepsilon^{\prime \prime }(\omega )$ with the real part $\varepsilon^{\prime }\left(\omega \right)$ and the imaginary part $\varepsilon^{\prime \prime }(\omega )$, the complex index of refraction is defined as $n^{*}=n+i\kappa$ with the real and the imaginary part n and κ (the extinction coefficient, giving rise to the damping of a light wave), respectively. With $\varepsilon_{r}\left(\omega \right)=(n^{*})2$, the relations $\varepsilon^{\prime }=n2-\kappa 2$ and $\varepsilon^{\prime \prime }=2n\kappa$ follow, where all parameters are frequency-dependent. Further calculations allow correlating n and κ with the conductivity of a material [81]. However, these calculations are scarcely performed in terms of nanofiber mats.

Nevertheless, the dielectric constant of nanofiber mats is often investigated, e.g., by impedance measurements, which enable calculating $\varepsilon^{\prime }\left(\omega \right)$ from the absolute value of the impedance or from an LCR meter with terminal parallel capacitance and $\varepsilon^{\prime \prime }\left(\omega \right)$ from the loss tangent [82,83,84]. However, only a few papers measured both optical refractive index values as well as dielectric constants [85,86,87], so that here only measurements of real and imaginary parts of the index of refraction are further described.

The extinction coefficient can be calculated by κ=αλ/(4π) with the absorption coefficient α and the light wavelength λ [88,89]. The absorption coefficient can be calculated from the measured absorbance by α=2.303A/d with the sample thickness *d* [90]. Kenawy et al. used this equation to determine the wavelength-dependent extinction coefficient of [1 H-Pyr+Ben]^B^ nanofiber thin film, which they suggested for energy storage and solar cell applications [91]. Going further, they calculated the real and imaginary part of the dielectric constant from *n* and κ and showed that the real part was much higher than the imaginary part, indicating that the examined nanofibers could store much electric and magnetic energy. Similarly, Matysiak and Tanski calculated the extinction coefficient of amorphous ZnO/crystalline ZnO NPs from the measured absorbance and showed a significant effect of the calcination temperature of the composite nanofibers on their optical properties [92]. Ibrahim et al. found a significantly increased extinction coefficient upon blending PEO with GO and multi-walled carbon nanotubes [93]. For PAN/PEO nanofibers with different amounts of GaN, Ahmad et al. calculated the extinction coefficient and refractive index and found a reduction in the extinction coefficient for wavelengths in the visible range of the spectrum, while the index of refraction increased [94].

The real part of the refractive index is more often mentioned than the extinction. This is probably due to the high impact of scattering in nanofiber mats, usually causing a low transparency [95]. Transparency is thus often significantly increased if the nanofiber mat is in a wet state, with water or other fluids filling the voids inside [96], or if nanomembranes with low fiber content are prepared by electrospinning instead of fully fibrous membranes [97]. In some cases, such as in the light scattering layers of dye-sensitized solar cells (DSSCs), this fact is even advantageous [98,99].

**Figure 4:**
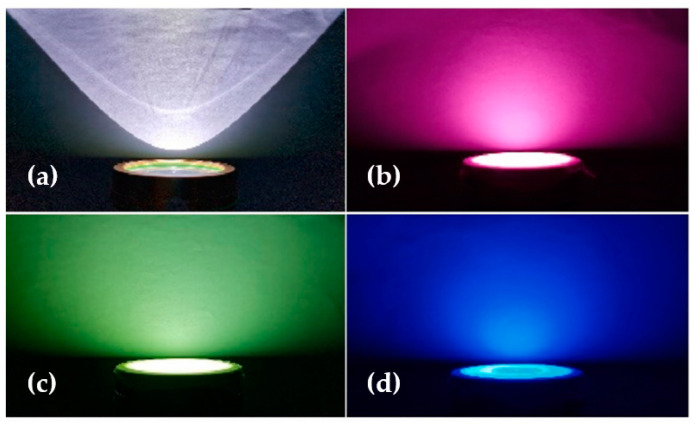
Comparison of (**a**) normal luminescence with (**b**) red, (**c**) yellow, and (**d**) blue colored scattered light of LED chip. From [101], copyright (2021), with permission from Elsevier.

While Fraunhofer diffraction is not applicable for nanofibers with diameters in the same order of magnitude as the impinging light wavelengths, the Mie theory—originally developed for small spherical particles—is often used with modifications, taking into account the anisotropic shapes of the nanofibers [100]. Based on the Mie scattering theory, Li et al. modeled light scattering from a nanofibrous PAN membrane and showed that light scattering properties could be adjusted by tailoring the nanofiber diameter, in this way improving the color yield and finally preparing LEDs with good light scattering characteristics [101]. As depicted in Figure 4, white luminescence of a commercial LED could be transferred into red, yellow, and blue luminescence by these nanofiber mats with tailored diameters, and at the same time, the light was scattered and thus protects the eye from the concentrated white light [101].

In addition to these applications where scattering was improved or reduced, partly wavelength-dependent, it is also possible to use measurements of scattering properties for sensing. In this way, Pirdadeh-Beiranvand et al. used resonance light scattering (RLS) of PVA nanofibers, decorated with Ni_0.5_Zn_0.5_Fe_2_O_4_ nanoparticles, to detect sunitinib, a cancer drug, from the change in the scattering intensity [102]. The authors also investigated the influence of diverse other ions or molecules on the change in the RLS spectra and found them to be negligible, i.e., that this optical sensor has a high selectivity towards sunitinib. Even more specialized sensing applications were reported for scattered reflection from magnetic nanofiber mats, usually referred to as D-MOKE (diffractive magneto-optical Kerr effect), where measurements along different angles of reflection were shown to contain information about the magnetism of a sample, which can be measured by measuring the rotation of the original linear polarization axis of the impinging laser beam [103,104].

## 6. Photoluminescence

Photoluminescence (PL) belongs to the often-mentioned optical properties of nanofiber mats [105,106,107] which are, however, not always fully defined. Photoluminescence can be differentiated into the fast fluorescence and the long-lasting phosphorescence, besides time-resolved measurement of photoluminescence, which measures the PL decay with time after excitation by a short light pulse. In all cases, the re-emitted photons are red-shifted, i.e., have smaller energy than the absorbed ones, often using UV irradiation to reach luminescence in the visible range. Among the typical applications of such measurements, there are investigations of semiconductors as well as fluorescence microscopic images of mammalian cells, stained with fluorescent dyes, allowing differentiation between the cell nucleus and fibroblasts [108,109,110]. Photoluminescence is also important for nanofiber-based solar concentrators with perovskites [111]. Perovskites can not only be used for solar cells [112,113,114] but may also show circularly polarized photoluminescence [115,116].

The photoluminescence spectrum, especially the maximum, can be influenced by the preparation of electrospun nanofiber mats, e.g., by the pyrolysis process of sulfur self-doped g-C_3_N_4_ nanofibers, which showed enhanced photocatalytic activity and light harvesting properties, as compared to the bulk material [117]. For electrospun bioactive glass containing Er^3+^ and Tb^3+^ ions, Deliormanli et al. found emission bands around 506 nm and 566 nm for an excitation wavelength around 374 nm, with additional bands for 1% Er^3+^ and more additional emission bands for Tb^3+^ or combinations of both dopants, showing different emission centers for the dopants [118]. Investigating different polymeric nanofibers with ZnO, Myundrul et al. found significantly increased photoluminescence of PVDF/ZnO nanofibers for an excitation wavelength of 325 nm [119].

In addition to these investigations of the sample properties by photoluminescence, PL can also be used, e.g., for oxygen sensing by praseodymium-modified ZnO nanofibers [120], while PSMMA nanofibers with LaPO_4_:Eu^3+^ were shown to have strong PL, making such nanofibers useful for optic and photonic devices [121]. Comparing undoped and Cu-doped ZnO electrospun nanofibers showed new defect states due to Cu incorporation into the ZnO lattice, resulting in polarized photoluminescence and decreased band gaps [122]. Packaging coaxially electrospun nanofibers with circularly polarized white luminescence with a UV chip resulted in white LEDs [123].

When a paper explicitly mentions fluorescence measurements, often optical sensors are the aim of the study [124]. Such electrospun fluorescence sensors can detect, e.g., ammonia [125], microRNA as a marker for cancer cells [126], nitroaromatic explosives [127], pH values [128], chiral recognition of molecular enantiomers [129], temperature [130], pathogenic bacteria [131], or aniline vapor [132].

Phosphorescence, on the other hand, was suggested as an alternative to electrical light in medical endoscopes [133] or for anti-counterfeiting applications [134], but in most cases it is used as an oxygen sensor [135,136,137,138,139]. Furthermore, other phosphorescent sensors were suggested, such as Zn^2+^ phosphorescent sensors [140,141], phosphorescent sensors for humidity [142], or several other physical and chemical stimuli, such as pressure, heat, pH value, explosives, or heavy metal ions [143].

## 7. Polarization

**Figure 5:**
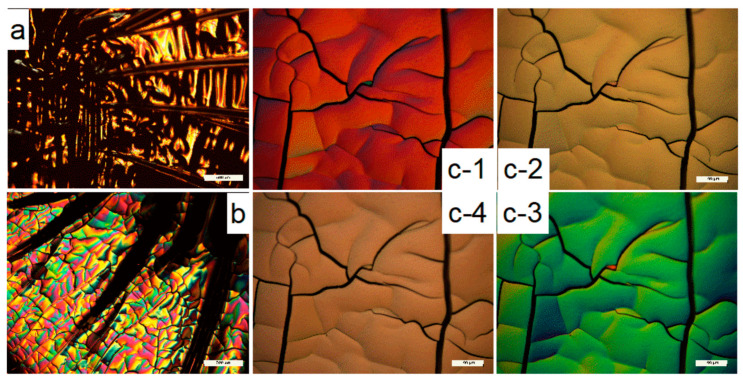
Polarized optical microscope images of lignin-based film without PEO and NCC and indicating different parts of the film have different optical properties (**a**) center, (**b**) edge of the film under lower magnification (×5, scale bar: 500 μm), and (**c-1**–**c-4**) edge part of the film under the higher magnification (×20, scale bar: 100 μm) with changing polarizer orientation from vertical (**c-1**) to lateral direction (**c-4**). Reprinted with permission from [144]. Copyright (2019) American Chemical Society.

In most cases, the aforementioned spectroscopic measurements are not related to the polarization of the incident or the emitted/reflected light. Investigations by polarized light optical microscopy, however, may be used to investigate the molecular orientation within the nanofibrous membrane, as depicted in Figure 5, where the edge of the film (Figure 5b) shows varying colors, and higher magnified micrographs taken with changing polarizer orientation (Figure 5(c-1–c-4)) indicate variation of the visible color [144]. While polarized measurements are also possible in the infrared [144,145], here we only discuss polarization in the visible range of the light spectrum [146].

Polarized optical microscopy was used, e.g., by Thum et al. who embedded liquid crystals (LCs) in coaxially electrospun nanofibers with a PVP sheath and azobenzene-doped LC core [147,148]. They showed that the azobenzene chromophores enabled photochemical switching between nematic and isotropic phases of the LC, i.e., UV light triggers the phase transition from nematic to isotropic phases, while visible light reversed this process. In this way, UV made the fibers visible in polarized optical microscopy (POM), and visible light made them invisible for the POM again.

Anisiei et al. used POM to roughly estimate the fiber crystallinity of chitosan/PEO and chitosan nanofibrous membranes [149]. They found birefringent textures in polarized light, which they identified as alignment of the chitosan chains upon electrospinning.

In addition to polarized optical microscopy, Bernardo et al. reported about polarized second harmonic generation (SHG) of anisotropic poly-ε-caprolactone (PCL) nanofibers in which nonlinear nanocrystals were embedded [150]. The setup to measure SHG was based on a mode-locked Ti:sapphire laser with a 100 fs pulse width and 76 MHz repetition rate. Variable polarizers were used in the incident and emitted light. The strong contrast in the measured curves at different polarizations revealed strong alignment of the nanocrystals inside the fibers.

Meng et al. embedded halide perovskite nanocrystals in PVA nanofibers and measured their polarization by using a picosecond pulsed diode laser whose light was circularly polarized as an incident beam, which was focused on the sample by an inverted fluorescence microscope [151]. The resulting photoluminescence from the sample was measured depending on these photons’ polarization. The authors found a strong polarization angle-dependence of the photoluminescence. By comparing experimental results and theoretical considerations, they attributed this effect to dielectric confinement with quantum confinement in the perovskite nanocrystals and suggested using such materials for displays, lasers, or waveguides. The same group also suggested nanofibers with embedded perovskite nanocrystals for down-shifting applications in liquid crystal display backlights or other polarization-dependent photonic devices [152].

Using an isotropic exciting light source, Chen et al. found strong anisotropy for emission with polarization parallel or perpendicular to the axis of their poly(3-hexylthiophene-2,5-diyl) (P3HT) nanofibers, with much higher emission intensity and a red-shifted maximum for the polarization perpendicular to the fiber axis [153].

Fu et al. showed by polarized luminescence measurements that CsPbBr_3_ nanorods embedded in polystyrene (PS) nanofibers could be aligned by an external magnetic field applied during electrospinning [154].

## 8. Bathochromic Shift

**Figure 6:**
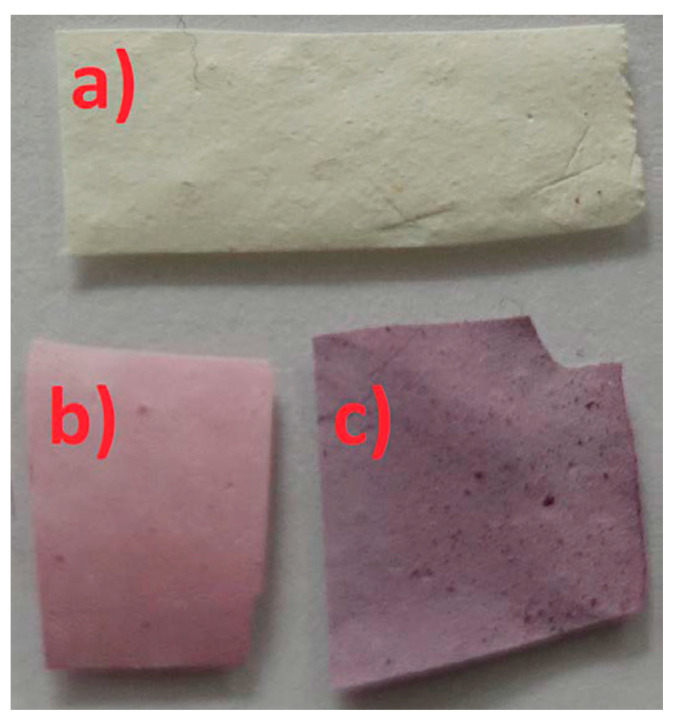
Photograph of differently treated electrospun nanofiber mats: (**a**) pure PAN-TiO_2_; (**b**) PAN after dip-coating in anthocyanin dye solution and drying; (**c**) PAN-TiO_2_ after dyeing, showing a bathochromic shift. From [158], copyright (2022), originally published under a CC-BY-SA license.

The bathochromic shift is a red-shift of the absorption spectrum of a material, based on the assembly between conjugated polymer backbones [155]. It is well known, e.g., from the binding of anthocyanins to TiO_2_ in DSSCs [156,157,158], as depicted in Figure 6 [158]. However, it can also be found in many other material blends or upon embedding nanoparticles in nanofibers [159,160,161].

Philip et al., e.g., reported bathochromic shifts when they embedded silver nanoparticles in poly(methyl methacrylate) (PMMA) nanofibers, which was attributed to the impact of the surface plasmon resonance absorption bands of the Ag NPs [162]. Similarly, Li et al. recognized a bathochromic shift when they compared the ternary (Eu(NTA)_3_L) (L = ligand) complex with Eu(NTA)_3_ embedded in PVA nanofibers, which they attributed to a coordination interaction with the ligand L [31]. Baptista et al. also reported a strong bathochromic shift between the photoluminescence spectra of 2-amino-4-nitroaniline and 3-nitroaniline nanocrystals embedded in poly-L-lactic acid nanofibers, besides SHG light emission [163].

On the other hand, Kato et al. used the bathochromic shift of the absorption maximum of PMMA upon contact with methanol solutions containing anions, which they attributed to the hydrogen bond formation between urea in the polymer and the anions penetrating into the nanofibers, thus using the bathochromic shift for sensing [164]. Similarly, Gal et al. found a strong bathochromic shift when their nanofiber mats containing zinc 10-ethyl-10*H*-phenothiazine-3carboxylate or rubidium 10-ethyl-10*H*-phenothiazine3-carboxylate got in contact with different solvents, also called solvatochromism [165].

**Figure 7:**
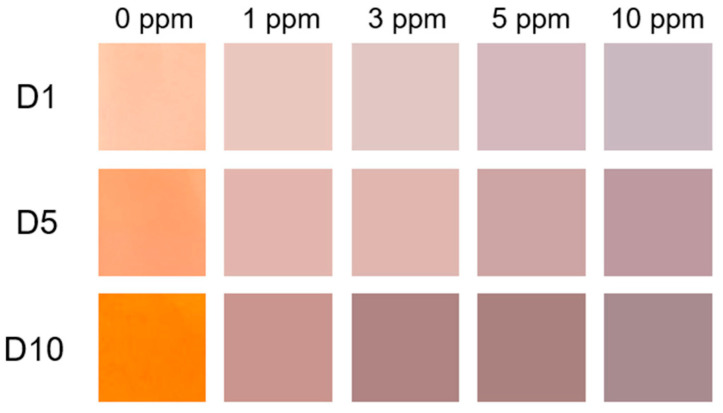
Color changes of each nanofiber sensor after exposure to various NH_3_ concentrations (1, 3, 5, and 10 ppm). Nanofibrous sensors D1, D5, and D10 contain 1%, 5%, and 10% of the dye, respectively. From [166], copyright (2020), originally published under a CC-BY license.

Oh et al. as well as Park et al. found bathochromic shifts of their meta-aramid/dye and cesium lead bromide perovskite nanofibrous membranes upon contact with ammonia, making these nanofiber mats suitable for ammonia sensing [166,167]. This effect is depicted in Figure 7 for varying NH_3_ concentrations [166].

## 9. Dyeing

In addition to the aforementioned bathochromic shift, based on chemical reactions, nanofiber mats can also change their color when they are dyed. An often-applied process is adding a dye directly to the spinning solution, which should result in a colored nanofiber mat [168,169,170,171].

For PVA nanofiber mats, Fadil added Remazol Yellow FG dye and Ase Direct Supra Red BWS dye to the spinning solutions, respectively, resulting in yellow and red dyed nanofibers, while the direct dye Ase Direct Supra Red BWS contained relatively large molecules and thus strongly increased the average fiber diameters [172].

**Figure 8:**
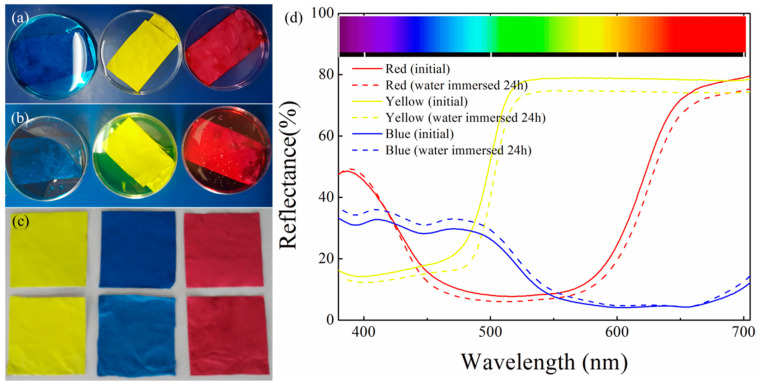
As-spun colored nanofibrous membranes (**a**) immersed in water; (**b**) after 24 h; (**c**) comparing membranes before and after immersion in water; and (**d**) UV-Vis diffuse reflectance spectroscopy of these membranes. From [173], copyright (2016), originally published under a CC-BY license.

Using cationic dyes, Yan et al. showed dyeing poly(vinyl butyral) (PVB) nanofiber mats in different colors, here red, yellow, light-blue, and black, which kept the colors nearly unchanged for 6 months [173]. Water-fastness of these colorful nanofiber mats is shown in Figure 8 [173].

Balakrishnan et al. chose alizarin, indigo, as well as different pigment dyes for dyeing PLA melt-electrospun nanofiber mats [174]. They reported a significant increase in the PLA melt viscosity, especially at low shear rates due to the added dyes; a decrease in the melt resistance; and colorant aggregates in some of the electrospun fibers. For the latter problem, they suggested integration of a screw system to provide shear during electrospinning.

Reactive dyes were used by Kishimoto et al. who dyed chitin nanofibers with blue, red, yellow colors, and mixtures of them, resulting in colors which were resistant even to hot water and detergents [175].

A combination of low IF emissivity and high solar absorption, useful for radiative heating of the human body in a cold environment, was reached by adding dyes to a PAN electrospinning solution, followed by magnetron sputtering a thin Ag layer on the electrospun nanofiber mat [176]. The IR emission could be controlled by tailoring the fiber diameter, while the Ag layer resulted in high solar radiation absorption.

Jatoi et al. chose another path and dyed electrospun PCL nanofiber mats in a continuous (pad-dry-cure) as well as a semicontinuous (pad-batch) process with disperse dyes [177]. They found good color fastness after washing, showing that dyeing after electrospinning is also possible. Similarly, PAN/CuS photothermal nanofiber mats, used for thermal management, were found to be dyeable [178]. Li et al. reported improved dyeability with anionic dyes when they modified PAN chemically before electrospinning [179].

## 10. Conclusions

While the optical properties of electrospun nanofiber mats are often ignored, in many cases they nevertheless enable investigations of their morphology, physical, and chemical properties. Transmission and absorbance can be tailored for the respective applications, while dyeing enables strong coloring in spite of the scattering of the mostly randomly oriented thin nanofibers. Furthermore, color changes of special nanofibrous materials can be used for sensing applications.

Modern research in the field of materials develops not only due to the achievement of greater resolving power of instruments measuring the electronic, mechanical, electrical, and optical parameters, but mainly thanks to the introduction of innovations to the inner structure of materials. Particularly important for this development is the transition from classical, structurally continuous materials, such as a variety of alloys, into structurally discontinuous materials. This results in completely new, sometimes surprising features. In the case of electrospun materials, the fibrous structure forces new properties. In the case of interactions between nanofiber mats and electromagnetic waves, local effects result from the existence of internal reflections and dispersion, which reveal specific dielectric and optical properties of the material as a whole. Similarly, in terms of electro-optical properties and the coupled elastic and thermal effects which are typical for electronic devices, phenomena such as reflection, transport through material interfaces, and conduction of light along single fibers occur, and at the same time, attempts to technologically control the anisotropy of optoelectronic properties on the macro scale. Appropriate selection of the production parameters of these nanofiber mats introduces components of directionality, resulting in anisotropic as well as optically and spectroscopically dispersive properties of these innovative materials.

While morphological or mechanical properties of electrospun nanofibrous membranes are often discussed, this review thus gives a brief overview of typical optical measurements which enable deeper characterization of nanofiber mats.

## Data Availability

No new data were created in this review paper.
